# Trends in the epidemiology of catheter-related bloodstream infections; towards a paradigm shift, Spain, 2007 to 2019

**DOI:** 10.2807/1560-7917.ES.2022.27.19.2100610

**Published:** 2022-05-12

**Authors:** Laia Badia-Cebada, Judit Peñafiel, Patrick Saliba, Marta Andrés, Jordi Càmara, Dolors Domenech, Emili Jiménez-Martínez, Anna Marrón, Encarna Moreno, Virginia Pomar, Montserrat Vaqué, Enric Limón, Úrsula Masats, Miquel Pujol, Oriol Gasch, on behalf of the VINCat programme (Infection Control Catalan Programme)

**Affiliations:** 1 Internal Medicine Department, Hospital Universitari Parc Taulí, Sabadell, Spain; 2 School of Medicine, Universitat Autònoma de Barcelona, Sabadell, Spain; 3 Unit of Statistics, Hospital Universitari de Bellvitge/Institut d'Investigació Biomèdica de Bellvitge-IDIBELL, L'Hospitalet de Llobregat, Spain; 4 VINCat programme: Infection Control Catalan Programme, Barcelona, Spain; 5 Infectious Diseases Unit, Department of Internal Medicine, Hospital Consorci de Terrassa, Spain; 6 Department of Microbiology, Hospital Universitari de Bellvitge, L'Hospitalet de Llobregat, Spain; 7 Institut d'Investigació Biomèdica de Bellvitge-IDIBELL, L'Hospitalet de Llobregat, Spain; 8 CIBER de Enfermedades Respiratorias (CIBERes), ISCIII, Madrid, Spain; 9 Infection Control Nurse, Hospital Josep Trueta, Girona, Spain; 10 Infection Control Nurse, Hospital Universitari de Bellvitge, Institut d'Investigació Biomèdica de Bellvitge-IDIBELL, L'Hospitalet de Llobregat, Spain; 11 Infectious Diseases Department, Hospital Universitari Parc Taulí, Sabadell, Spain; 12 Infection Control Nurse, Parc Sanitari Sant Joan de Déu, Sant Boi de Llobregat, Spain; 13 Infectious Diseases Unit, Department of Internal Medicine, Hospital de la Santa Creu I Sant Pau, Barcelona, Spain; 14 Infection Control Nurse, Hospital de Barcelona, Barcelona, Spain; 15 Infection Control Nurse, Hospital Universitari Mútua Terrassa, Terrassa, Spain; 16 Department of Infectious diseases, Hospital Universitari de Bellvitge, L'Hospitalet de Llobregat, Spain; 17 Institut d'Investigació Biomèdica de Bellvitge-IDIBELL, L'Hospitalet de Llobregat, Spain; 18 Institut d’Investigació i Innovació Parc Taulí, Sabadell, Spain; 19 The members of the network are acknowledged at the end of the article

**Keywords:** catheter-related bloodstream infection, epidemiology, bundle, peripheral catheter, nosocomial infection

## Abstract

**Background:**

Catheter-related bloodstream infections (CRBSI) are frequent healthcare-associated infections and an important cause of death.

**Aim:**

To analyse changes in CRBSI epidemiology observed by the Infection Control Catalan Programme (VINCat).

**Methods:**

A cohort study including all hospital-acquired CRBSI episodes diagnosed at 55 hospitals (2007–2019) in Catalonia, Spain, was prospectively conducted. CRBSI incidence rates were adjusted per 1,000 patient days. To assess the CRBSI rate trend per year, negative binomial models were used, with the number of events as the dependent variable, and the year as the main independent variable. From each model, the annual rate of CRBSI diagnosed per 1,000 patient days and the incidence rate ratio (IRR) with its 95% confidence intervals (CI) were reported.

**Results:**

During the study, 9,290 CRBSI episodes were diagnosed (mean annual incidence rate: 0.20 episodes/1,000 patient days). Patients’ median age was 64.1 years; 36.6% (3,403/9,290) were female. In total, 73.7% (n = 6,845) of CRBSI occurred in non-intensive care unit (ICU) wards, 62.7% (n = 5,822) were related to central venous catheter (CVC), 24.1% (n = 2,236) to peripheral venous catheters (PVC) and 13.3% (n = 1,232) to peripherally-inserted central venous catheters (PICVC). Incidence rate fell over the study period (IRR: 0.94; 95%CI: 0.93–0.96), especially in the ICU (IRR: 0.88; 95%CI: 0.87–0.89). As a whole, while episodes of CVC CRBSI fell significantly (IRR: 0.88; 95%CI: 0.87–0.91), peripherally-inserted catheter CRBSI (PVC and PICVC) rose, especially in medical wards (IRR PICVC: 1.08; 95%CI: 1.05–1.11; IRR PVC: 1.03; 95% 1.00-1.05).

**Conclusions:**

Over the study, CRBSIs associated with CVC and diagnosed in ICUs decreased while episodes in conventional wards involving peripherally-inserted catheters increased. Hospitals should implement preventive measures in conventional wards.

## Introduction

The use of vascular devices in hospitalised patients is essential for their treatment, which frequently involves the administration of drugs and fluids, parenteral nutrition, or haemodialysis. The prevalence of peripheral (PVC) and central (CVC) venous catheter use among hospitalised patients estimated in different European surveys in the last decade is around 70% and 10% respectively [1-3]. In in a prospective cohort study published in 2010, catheter-related bloodstream infections (CRBSI) were the most important complications reported from 15 Spanish hospitals, with 821 bloodstream infections (BSI) episodes, representing almost 25% of all nosocomial BSI [4]. According to a paper from 2006 reporting a systematic review of 200 published prospective studies, the incidence rate of CRBSI per 1,000 catheter days generally ranges from 0.1 episodes for PVC to 2.7 episodes for CVC [5].

CRBSI are an important cause of morbidity and mortality. Patients with these infections usually have more severe underlying illness and are more likely to have other healthcare-associated infections (HAI) during their admission, with a mortality ranging from 12% to 25%, according to a prospective nationwide surveillance study in the United States (US) from March 1995 through September 2002 [6]. CRBSI are also associated with longer hospital admissions and higher economic costs [7].

The application of prevention programmes in intensive care units (ICU) in recent decades has resulted in significant reductions of CRBSI incidence rates [8]. Bundles of preventive measures have been applied including hand hygiene, use of chlorhexidine alcohol solution for skin antisepsis, full barrier precautions, daily review of need for catheterisation and femoral site avoidance [8].

The Infection Control Catalan Programme (VINCat) was launched in 2006, with the main objective of reducing the incidence of HAI through continuous active monitoring and implementation of preventive programmes. Surveillance of CRBSI at the hospitals in our region is a priority [9]. The aim of this study is to describe the changes in the incidence and epidemiology of CRBSI in the hospitals participating in the VINCat programme over a 13-year period.

## Methods

### Setting

BSI associated with the use of venous catheters is continuously monitored under the VINCat programme. Participation of hospitals in VINCat is voluntary. All detected nosocomial episodes of CRBSI, which are diagnosed in adult patients at each of the participating hospitals are prospectively followed and reported to the VINCat programme by the infection control teams. The detection of cases is based on the daily evaluation of all patients with positive blood cultures. This information is provided to the infection control team by the microbiology laboratory at each hospital. The application of precise definitions allows the identification of CRBSI.

The 55 Catalan hospitals participating in the VINCat programme are classified into three categories according to complexity and to the number of beds available for hospitalisation: 500 beds or more (Group I), 200 to 499 beds (Group II), and fewer than 200 beds (Group III). A table with the number of hospitals participating in each year of the study, stratified by group, is provided in Supplement S1, with a footnote providing further details on the function of these hospitals.

Data from each hospital are continuously monitored. The annual incidence rates are compared with the hospitals’ records from previous years, and with the aggregate data compiled in the VINCat programme. Results are presented at general clinical sessions and a public annual report is published within the VINCat website [9]. This study presents data from all episodes recorded between January 2007 and December 2019.

### Definitions

Terms used in this study are described as follows [10].

#### Catheter-related bloodstream infection

A bacterial infection in a patient using a venous catheter is defined with the following criteria. It has to be detected with at least one set of blood cultures obtained from a peripheral vein and two sets in the case of habitual skin-colonising microorganisms (coagulase-negative *staphylococci* (CoNS), *Micrococcus* spp., *Propionibacterium acnes*, *Bacillus* spp. and *Corynebacterium* spp.). These cultures must be associated with clinical manifestations of infection (fever > 37.5 °C, chills and/or hypotension) and the absence of any apparent alternative source of BSI.

These conditions must be accompanied by one or more of the following:

(i) semiquantitative culture of catheter tip (> 15 colony forming units (CFU) per catheter segment) or quantitative culture (> 10^3^ CFU per catheter segment), with detection of the same microorganism as in blood cultures obtained from the peripheral blood;

(ii) quantitative blood cultures with detection of the same microorganism, with a difference of 5:1 or greater between the blood obtained from any of the lumens of a venous catheter and that obtained from a peripheral vein by puncture;

(iii) difference in time to positivity of the blood cultures of above 2 hours between cultures obtained from a peripheral vein and from the lumen of a venous catheter;

(iv) presence of inflammatory signs or purulent secretions in the insertion point or the subcutaneous tunnel of a venous catheter. A culture of the secretion showing growth of the same microorganism as the one detected in the blood cultures is also recommended (but not obligatory);

(v) resolution of clinical signs and symptoms after catheter withdrawal with or without appropriate antibiotic treatment. For the clinical diagnosis of PVC-BSI, the presence of signs of phlebitis is required (induration, pain or signs of inflammation at the insertion point or the catheter route). This last criterion is the only one that is not considered in the point prevalence survey protocol for microbiology confirmed catheter-related infection (CRI3-CVC) [11].

#### Type of catheter

A CVC is defined as a catheter inserted in a subclavian, jugular or femoral vein, percutaneously (with or without tunneling). Fully implanted catheters (type Port-a-Cath) are not included in the surveillance programme. A peripherally-inserted central venous catheter (PICVC) is a catheter inserted percutaneously through a vein in the forearm (usually a basilica vein). Its distal end reaches the right heart cavities. These catheters are generally used in the same way as conventional CVCs. A PVC is a short- or medium-length catheter inserted percutaneously in a peripheral location (usually an arm or forearm).

#### Hospital wards

Hospital wards where CRBSI are identified are classified as medical, surgical, or ICUs.

### Exclusion criteria

Episodes in the following patients were not included in the study: patients up to 18 years of age; outpatients with a hospital stay of less than 48 hours at time of BSI detection; patients in whom CRBSI was detected at an outpatient service; CRBSI associated with arterial catheters.

### Statistical analysis

Categorical variables were presented as the number of cases and percentages. Continuous variables were presented as means and standard deviation (SD) or medians and interquartile range (IQR), depending on whether the distribution was normal or non-normal. Normality of variables was assessed graphically (quantile-quantile-plot and density plots).

The annual incidence rate of CRBSI was obtained by dividing the total number of episodes of CRBSI with the total number of patient days in 1 year and this was then adjusted for 1,000 patient days to give the annual incidence rate of CRBSI diagnosed per 1,000 patient days (annual incidence of CRBSI diagnosed per 1,000 patient days  = total number of CRBSIs detected in 1 year x 1,000 /number of patient days).

A negative binomial model was used to assess the trend over the study period of the rate CRBSIs diagnosed at VINCat hospitals per year. The number of admissions per year was used as offset, the number of events (i.e. CRBSI) as the dependent variable, and the year as the main independent variable. The effect of hospital ward, catheter type and the interaction between year and catheter type, catheter use and aetiology were also assessed. Stratified analysis according to hospital ward and catheter type was also performed. From each scenario, the annual incidence rate of CRBSIs diagnosed per 1,000 patient days was reported, as was the incidence rate ratio (IRR) with its 95% confidence interval (CI). The interpretation of IRR was focused on the annual rate increase or decrease. The expected annual numbers of CRBSIs were plotted.

To estimate catheter days, we obtained the total adult patient days from all the centres during the study period and then multiplied this total by the mean prevalence rate of catheter daily use over this period [12]. All analyses were performed with a two-sided significance level of 0.05 and conducted with the R software version 4.0.2 [13].

### Microbiology

Two sets of two blood samples from a peripheral vein are usually obtained from all patients with a suspected BSI. An additional blood sample is also collected through the catheter. When possible, the catheter tip is cultured after removal. Blood samples are processed at the microbiology laboratories of each centre in accordance with standard operating procedures. Every microorganism is identified using standard microbiological techniques at each centre.

## Results

During the study period, a total of 9,290 CRBSI episodes were reported (Table 1). The incidence rate was 0.20 episodes/1,000 patient days. Patients’ median age was 64.1 years, and 36.6% of patients were female (information on sex was collected as a binary variable). BSI was diagnosed a median of 10 days (IQR: 6–17) after admission and a median of 3 days (IQR: 0–14) after catheter insertion. In total, 26.3% of episodes occurred in the ICU, while 42.1% and 31.6% were acquired in medical and surgical wards, respectively. Among the whole cohort, 62.7% episodes were related to CVC, 24.1% to PVC and 13.3% to PICVC, while catheter use was distributed as haemodialysis (4.8%), parenteral nutrition (26.6%) and other uses (68.7%). Meanwhile, the most frequent responsible microorganisms were CoNS (39.5%), followed by *Staphylococcus aureus* (24.6%) and *Enterobacteriaceae* (18.4%). *Candida* species accounted for 5.9% episodes, *Pseudomonas aeruginosa* 5.2% and *Enterococcus* spp. 5.0%.

**Table 1:** Clinical and demographic characteristics of annual catheter-related bloodstream infections diagnosed at VINCat hospitals, Catalonia, Spain, 2007–2019 (n = 9,290)

Characteristics	ALL (n = 9,290)		2007 (n = 741)		2008 (n = 784)		2009 (n = 834)		2010 (n = 775)		2011 (n = 896)		2012 (n = 752)			2013 (n = 588)			2014 (n = 694)		2015 (n = 703)		2016 (n = 678)			2017 (n = 619)		2018 (n = 688)		2019 (n = 538)	
	n^a^	%^b^	n^a^	%^b^	n^a^	%^b^	n^a^	%^b^	n^a^	%^b^	n^a^	%^b^	n^a^		%^b^	n^a^		%^b^	n^a^	%^b^	n^a^	%^b^	n^a^		%^b^	n^a^	%	n^a^	%^b^	n^a^	%^b^
**Patient**																															
Age, mean (SD)	64.1 (15.7)		62.2 (16.2)		61.9 (15.8)		64.0 (16.2)		63.7 (15.5)		63.1 (16.0)		64.4 (15.4)			64.2 (14.8)			64.5 (15.8)		64.6 (15)		65.9 (14.9)			65.2 (15.6)		65.0 (15.4)		65.4 (16.4)	
Female sex^c^	3,403	36.6	286	38.6	315	40.2	320	38.4	311	40.1	320	35.7	264	35.1		206	35.0		237	34.1	240	34.1	244	36.0		208	33.6	245	35.6	207	38.5
**Catheter**																															
Days since catheter insertion, Md (IQR)	3.0 (0.0–14.0)		4.0 (0.0–17.5)		5.0 (1.0–19.0)		5.0 (1.0–18.0)		3.0 (0.0–12.0)		3.0 (0.0–14.0)		3.0 (0.0–13.0)			3.0 (0.0–13.0)			3.0 (0.0–14.0)		4.0 (0.0–13.0)		2.0 (0.0–12.8)			3.0 (0.0–12.0)		3.0 (0.0–13.0)		3.0 (0.0–10.0)	
Days since admission, Md (IQR)	10.0 (6.0–17.0)		10.0 (6.0–17.0)		11 (7.0–17.0)		10.0 (6.0–18.0)		10.0 (6.0–17.0)		10.0 (5.3–18.0)		9.0 (6.0–16.0)			10.0 (6.0–18.0)			10.0 (5.0–17.0)		10.0 (6.0–18.0)		10.0 (6.0–18.0)			9.0 (6.0–18.0)		9.0 (5.0–17.0)		8.0 (5.0–16.0)	
**Catheter type**																															
CVC	5,822	62.7	541	73.0	616	78.6	608	72.9	558	72.0	605	67.5	447		59.4	386		65.6	417	60.1	423	60.2	362		53.4	321	51.9	319	46.4	219	40.7
PVC	2,236	24.1	137	18.5	117	14.9	151	18.1	148	19.1	205	22.9	207		27.5	127		21.6	160	23.1	188	26.7	179		26.4	179	28.9	237	34.4	201	37.4
PICVC	1,232	13.3	63	8.5	51	6.5	75	8.9	69	8.9	86	9.6	98		13.0	75		12.8	117	16.9	92	13.1	137		20.2	119	19.2	132	19.2	118	21.9
**Site of acquisition**																															
Medical ward	3,911	42.1	262	35.4	304	38.8	318	38.1	324	41.8	397	44.3	318		42.3	242		41.2	268	38.6	297	42.2	279		41.2	284	45.9	345	50.1	273	50.7
Surgical ward	2,934	31.6	242	32.7	217	27.7	264	31.7	231	29.8	247	27.6	238		31.6	184		31.3	245	35.3	240	34.1	238		35.1	208	33.6	213	31.0	167	31.0
ICU	2,445	26.3	237	32	263	33.5	252	30.2	220	28.4	252	28.1	196		26.1	162		27.6	181	26.1	166	23.6	161		23.7	127	20.5	130	18.9	98	18.2
**Catheter use**																															
Haemodialysis	444	4.8	37	4.9	52	6.6	46	5.5	64	8.3	60	6.7	34		4.5	30		5.1	25	3.6	25	3.6	23		3.4	16	2.6	21	3.1	11	2.0
PN	2,467	26.6	202	27.3	216	27.6	253	30.3	214	27.6	219	24.4	195		25.9	147		25	180	25.9	204	29	181		26.7	165	26.7	177	25.7	114	21.2
Other	6,379	68.7	502	67.7	516	65.8	535	64.1	497	64.1	617	68.9	523		69.5	411		69.9	489	70.5	474	67.4	474		69.9	438	70.8	490	71.2	413	76.8
**Aetiology^d^**																															
CoNS	3,652	39.5	345	46.6	343	43.8	368	44.3	316	40.8	328	36.8	286		38.0	233		39.6	248	35.7	250	35.6	268		39.6	259	42.1	234	34.9	174	33.6
*S. aureus*	2,268	24.6	153	20.7	165	21.0	176	21.2	146	18.9	201	22.5	166		22.1	137		23.3	186	26.8	190	27.1	165		24.4	156	25.4	229	34.2	198	38.2
*Enterobacteriaceae*	1,700	18.4	110	14.9	134	17.1	153	18.4	157	20.3	183	20.5	157		20.9	104		17.7	131	18.9	137	19.5	131		19.4	109	17.7	111	16.6	83	16
*Enterococcus* spp.	459	5.0	46	6.2	29	3.7	34	4.1	49	6.3	58	6.5	49		6.5	39		6.6	41	5.9	31	4.4	22		3.3	18	2.9	27	4.0	16	3.1
*Candida* spp.	540	5.9	41	5.5	45	5.7	51	6.1	45	5.8	56	6.3	43		5.7	34		5.8	42	6.1	42	5.9	47		6.9	36	5.8	31	4.6	27	5.2
*P. aeruginosa*	483	5.2	36	4.9	46	5.9	30	3.6	50	6.5	55	6.2	44		5.8	39		6.6	38	5.5	48	6.8	36		5.3	27	4.4	20	2.9	14	2.7
Other	135	1.5	9	1.2	22	2.8	19	2.9	11	1.4	11	1.2	7		0.9	2		0.3	8	1.2	4	0.6	8		1.2	10	1.6	18	2.7	6	1.2

CoNS: coagulase-negative staphylococci; CVC: central venous catheter; ICU: intensive care unit; IQR: interquartile range; Md: median; *P. aeruginosa*: *Pseudomonas aeruginosa*; PICVC: peripherally-inserted central venous catheter; PN: parenteral nutrition; PVC: peripheral venous catheter; *S. aureus*: *Staphylococcus aureus*; SD: standard deviation; VINCat: Infection Control Catalan Programme.

^a^ Numbers of counts are presented in this column, unless otherwise specified by the row header.

^b^ Percentages are presented in this column, unless otherwise specified by the row header.

^c^ Information on sex was collected as a binary variable.

^d^ Data on aetiology were missing for 53 catheter-related bloodstream infections, including one in 2007, three in 2009, one in 2010, four in 2011, one in 2015, one in 2016, four in 2017, 18 in 2018 and 20 in 2019.

### Annual incidence trends

**Figure 1:**
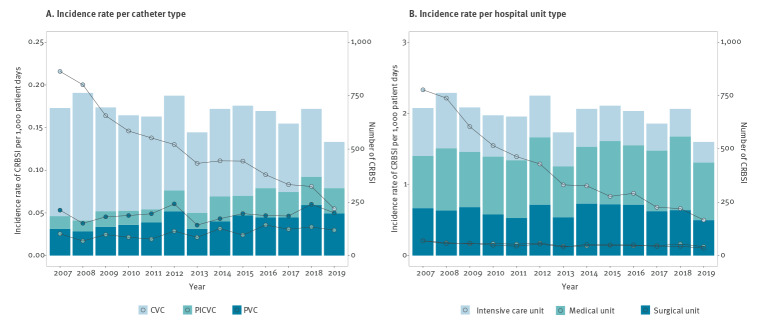
Annual incidence rate of catheter-related bloodstream infection adjusted per 1,000 patient days stratified by (A) catheter type and (B) hospital unit type, Catalonia, Spain, 2007–2019 (n = 9,290)

CRBSI: catheter-related bloodstream infection; CVC: central venous catheter; PIVC: peripherally-inserted central venous catheters; PVC: peripheral venous catheters.

The annual incidence rate of CRBSI fell from 0.29 episodes per 1,000 patient days in 2007 to 0.13 in 2019 (IRR: 0.94; 95%CI: 0.93–0.96) (Table 2). This downward trend was mostly associated with the progressive decrease in the annual incidence rate of CVC CRBSI, which ranged from 0.22 per 1,000 patient days in 2007 (507 episodes) to 0.05 (217 episodes) in 2019 (Figure 1A). A downward trend in CRBSI episodes acquired in the ICU was also observed during the study period, from 2.33 to 0.5 episodes/1,000 patient days (IRR: 0.88; 95%CI: 0.87–0.89), while the annual incidence rate of episodes acquired in medical and surgical wards presented significantly lower decreases (IRR: 0.97; 95%CI: 0.96–0.98 and IRR: 0.97; 95%CI: 0.95–0.98, respectively). Incidence rates of episodes acquired in the ICU, regardless of catheter type (CVC, PVC or PICVC), followed a downward trend. Meanwhile, in the medical wards annual incidence rates of CVC BSI fell significantly (IRR: 0.90; 95%CI: 0.89–0.92) but those of PVC and PICVC increased (IRR: 1.03; 95%CI: 1.00–1.05 and IRR: 1.08; 95%CI: 1.05–1.11, respectively). In the surgical wards, CVC episodes fell significantly (IRR: 0.94; 95%CI: 0.93–0.96) while CRBSI associated with PICVC increased (IRR: 1.05; 95%CI: 1.01–1.09) (Figure 1B, Table 2).

**Table 2:** Annual incidence rate per 1,000 patient days of catheter-related bloodstream infections diagnosed at VINCat hospitals stratifying by hospital ward and catheter type, Catalonia, Spain, 2007–2019 (n = 9,290)

Variables		Incidence^a^													IRR (95%CI)^c^
		2007	2008	2009	2010	2011	2012	2013	2014	2015	2016	2017	2018	2019	
**Number of CRBSI episodes^b^**		692	763	695	658	652	750	578	688	703	678	619	688	533	
**Number of patient days**		2,347.947	2,991,053	2,970,611	3,067,156	3,161,235	3,416,998	3,497,772	3,700,237	3,818,378	3,817,357	3,855,069	3,936,649	3,953,391	
**ICU**	**CVC**	2.01	1.96	1.48	1.3	1.15	1.06	0.78	0.77	0.62	0.63	0.52	0.46	0.34	0.87 (0.86–0.88)
	**PVC**	0.09	0.14	0.13	0.06	0.06	0.09	0.09	0.03	0.07	0.07	0.03	0.07	0.04	0.92 (0.88–0.96)
	**PICVC**	0.23	0.12	0.20	0.18	0.18	0.14	0.13	0.18	0.15	0.17	0.13	0.13	0.13	0.97 (0.94–1)
	**Subtotal**	2.33	2.22	1.81	1.55	1.39	1.29	1.0	0.98	0.83	0.88	0.68	0.66	0.50	0.88 (0.87–0.89)
**Medical wards**	**CVC**	0.14	0.13	0.1	0.1	0.08	0.08	0.07	0.06	0.06	0.05	0.05	0.05	0.03	0.90 (0.89–0.92)
	**PVC**	0.06	0.04	0.06	0.06	0.07	0.08	0.05	0.06	0.07	0.06	0.06	0.09	0.07	1.03 (1.00 –1.05)
	**PICVC**	0.01	0.01	0.01	0.01	0.01	0.02	0.01	0.02	0.02	0.02	0.03	0.03	0.02	1.08 (1.05–1.11)
	**Subtotal**	0.21	0.18	0.17	0.17	0.16	0.18	0.13	0.14	0.15	0.14	0.14	0.16	0.13	0.97 (0.96–0.98)
**Surgical wards**	**CVC**	0.14	0.13	0.12	0.10	0.10	0.10	0.09	0.10	0.11	0.09	0.07	0.07	0.06	0.94 (0.93–0.96)
	**PVC**	0.05	0.02	0.02	0.03	0.02	0.03	0.01	0.03	0.03	0.03	0.03	0.03	0.02	0.98 (0.94–1.02)
	**PICVC**	0.02	0.01	0.03	0.01	0.01	0.03	0.02	0.03	0.02	0.04	0.03	0.03	0.03	1.05 (1.01–1.09)
	**Subtotal**	0.21	0.17	0.18	0.14	0.13	0.16	0.12	0.16	0.15	0.15	0.13	0.13	0.10	0.97 (0.95–0.98)
**Total**		0.29	0.26	0.23	0.21	0.21	0.22	0.17	0.19	0.18	0.18	0.16	0.17	0.13	0.94 (0.93–0.96)

CI: confidence interval; CRBSI: catheter-related bloodstream infection; CVC: central venous catheter; ICU: intensive care unit; IRR: incidence rate ratio; PVC: peripheral venous catheter; PICVC: peripherally-inserted central venous catheter; VINCat: Infection Control Catalan Programme.

^a^ Incidence rates per 1,000 patient days for each year of the study are presented in the columns, unless otherwise specified by the row header.

^b^ Episodes diagnosed in hospitals, where the number of patient days were available.

^c^ Incidence risk ratio by year (with the inclusion of CRBSI episodes diagnosed in hospitals, for which patient days were available that year).

An estimation of incidence rate of CRBSI adjusted per 1,000 catheter days was carried out to overcome possible bias associated with catheter use, and a significant downward trend in the incidence rates of CRBSI associated to CVC was also observed, both in ICUs and conventional wards (Supplement S1).

### Interaction between years and episode characteristics

**Figure 2:**
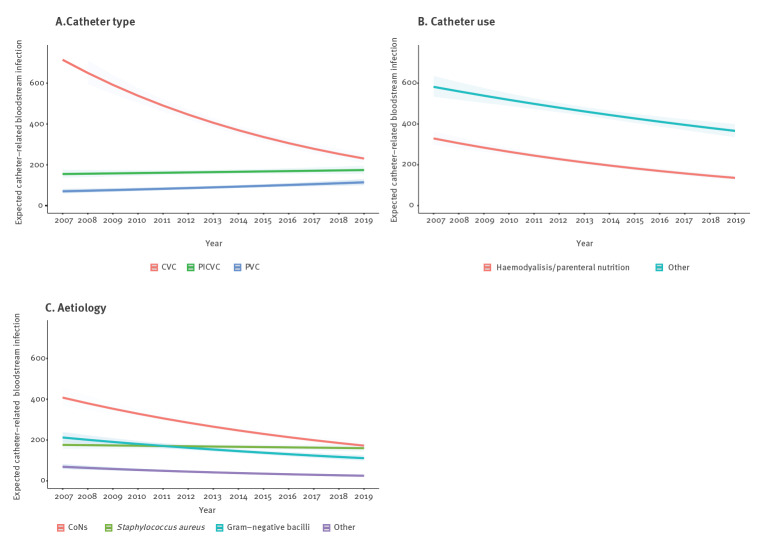
Number of expected catheter-related bloodstream infection per year, stratified by (A) catheter type, (B) catheter use and (C) aetiology, Catalonia, Spain, 2007–2019 (n = 9,290)

CoNS: coagulase-negative staphylococci; CVC: central venous catheter; PIVC: peripherally-inserted central venous catheters; PVC: peripheral venous catheters.

**Figure 3:**
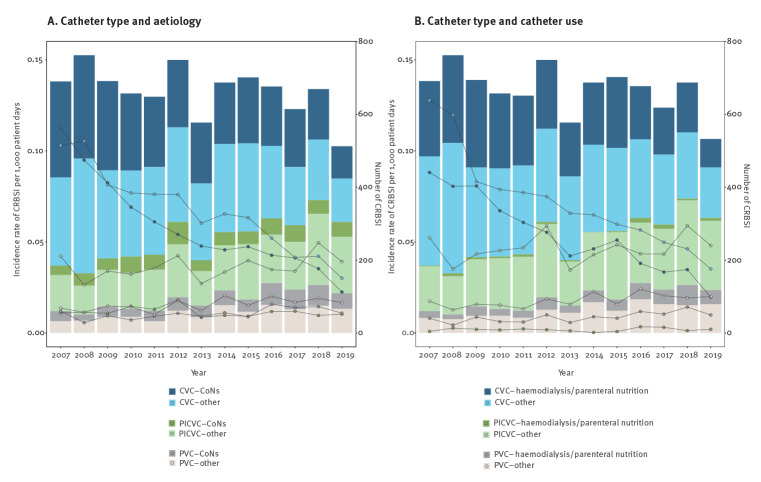
Annual incidence rate of catheter-related bloodstream infection adjusted per 1,000 patient days stratified by (A) catheter type and aetiology and (B) catheter type and use, Catalonia, Spain, 2007–2019 (n = 9,290)

CRBSI: catheter-related bloodstream infection; CoNS: coagulase-negative staphylococci; CVC: central venous catheter; PICVC: peripherally-inserted central venous catheter; PVC: peripheral venous catheter.

The interactions between year and catheter type or catheter use were respectively statistically significant (Figure 2A, Figure 2B, Table3). For catheter use, significance was observed despite the absolute number of episodes of all three categories (parenteral nutrition, haemodialysis and other uses) falling during the study period. On the other hand, the category of ‘other uses’ increased compared with the other two (Table 3). The interaction between year and aetiology was statistically significant, with a significant downward trend in the rate of episodes caused by CoNS. Simultaneously, incidence rates of *S. aureus* rose significantly (Figure 2C, Table3). Figure 3 shows the annual incidence rate of CRBSI adjusted per 1,000 patient days stratified by catheter type, catheter use and microorganism.

Gram-negative bacilli include *Enterobacteriaceae* and *Pseudomonas aeruginosa.*

**Table 3:** Negative binomial model with interaction between years and episode characteristics, Catalonia, Spain, 2007–2019 (n = 9,290)

Predictors	IRR	CI	p
**Interaction between years and catheter types **			
**Intercept**	0.00	0.00–0.00	< 0.001
**Year**	0.91	0.90–0.92	< 0.001
PICVC vs CVC	0.20	0.16–0.23	< 0.001
PVC vs CVC	0.09	0.07–0.11	< 0.001
Interaction between PICVC and year	1.11	1.08–1.14	< 0.001
Interaction between PVC and year	1.14	1.12–1.17	< 0.001
**Interaction between years and catheter use**			
**Intercept**	0.00	0.00–0.00	< 0.001
**Year**	0.93	0.92–0.94	< 0.001
Other uses vs haemodialysis/PN	1.71	1.47–1.98	< 0.001
Interaction between other uses and year	1.04	1.02–1.06	< 0.001
**Interaction between years and aetiology**			
**Intercept**	0.00	0.00–0.00	< 0.001
**Year**	0.93	0.92–0.95	< 0.001
*Staphylococcus aureus* vs CoNS	0.41	0.34–0.49	< 0.001
Gram-negative bacilli vs CoNS	0.51	0.43–0.61	< 0.001
Other vs CoNS	0.17	0.14–0.22	< 0.001
Interaction between *Staphylococcus aureus* and year	1.07	1.04–1.09	< 0.001
Interaction between Gram-negative bacteria and year	1.02	0.99–1.04	0.138
Interaction between other pathogens and year	0.99	0.96–1.02	0.405

CI: confidence interval; CoNS: coagulase-negative staphylococci; CVC: central venous catheter; IRR: incidence rates ratios; PICVC: peripherally-inserted central venous catheter; PN: parenteral nutrition; PVC: peripheral venous catheter.

Gram-negative bacilli include *Enterobacteriaceae* and *Pseudomonas aeruginosa.*

## Discussion

This study is a comprehensive description of the changes in the epidemiology of CRBSI at hospitals in our healthcare region in Spain. Our large series of CRBSI reveals a significant increase in the incidence rate of PVC and PICVC CRBSI in conventional wards, in parallel to a notable reduction in all CRBSI in ICUs.

During the study period, three interventional programmes were implemented in hospitals belonging to the VINCat. First, since 2007 the comparative results (benchmarking) of CRBSI surveillance are shared with professionals involved in prevention of complications associated with vascular catheters [14]. As was already demonstrated in 1985 by a study in the US, implementation of intensive infection surveillance and control programmes can reduce the rate of nosocomial infections by up to 30% [15].

The second intervention implemented at our hospitals was the so-called ‘Bacteraemia Zero Programme’. It started in 2009 at 192 Spanish ICUs, including all in hospitals in Group I and resulted in a reduction in the risk of CRBSI of 50% in 18 months [16]. Consistently, in recent years, prevention programmes in ICUs have enabled to bring down CVC CRBSI incidence rates. One of the most influential of these programmes was implemented in 2004 in 103 ICUs from 67 Michigan hospitals, as described by Pronovost et al. [8]. These authors reported a 66% reduction in CRBSI rates, which fell to zero infections per 1,000 catheter days 18 months after the programme start [8]. A meta-analysis including 43 studies, published in 2014, also showed a decrease in CVC CRBSI incidence rates after the implementation of prevention programmes in ICUs [17]. Therefore, the impact of these programmes in ICUs is beyond doubt.

The third intervention implemented in VINCat hospitals was conducted in 2010 and included the monitoring of CRBSI related to all PVC and CVC catheters inserted at conventional wards of 11 hospitals of the VINCat programme [18]. Studies assessing the incidence rate of CRBSI in conventional wards compared to ICU present a wide range of results [19-21]. In our study, the incidence rate of CVC CRBSI in the ICU was higher than that observed in conventional settings, but the difference between the rates fell over the course of the study period. In the present study we also found an upward trend in the PVC CRBSI incidence rate in medical wards, in agreement with recent prospective studies [22-25].

Our results suggest the need for programmes in conventional wards to prevent PVC CRBSI, similar to the ones conducted in ICUs in recent years. Indeed, a multimodal intervention performed in conventional wards of a selection of Spanish hospitals was associated with a reduction of CVC CRBSI incidence in this setting. However, no impact on PVC CRBSI incidence was observed. It was concluded that compliance with the bundles related to catheter insertion and maintenance was lower for PVCs, probably due to the lower perception of risk of complications with their use [18]. However, we stress that other similar experiences achieved significant reductions in PVC CRBSI incidence rates in conventional settings [26-28].

In this study, the incidence rate of CRBSI caused by CoNS followed a downward trend, in agreement with a previous report [29]. In contrast, it was observed that, relative to the CoNS, the Gram-negative bacilli incidence increased, as recently described elsewhere [25,30]; the risk was associated with solid organ transplantation, prior use of antibiotics, previous neurological or gastrointestinal conditions, and longer hospital stay [25,29,31]. Although femoral catheters have also been associated with higher infection rates due to Gram-negative bacilli [8,32], we did not observe this relationship (data not shown), perhaps because prevention programmes in recent years have argued against their use and their insertion is less frequent today than in the past [33]. Other studies have underlined the importance of hand hygiene to prevent CRBSI caused by Gram-negative bacteria [28].

Similarly, *S. aureus* CRBSI episodes also increased during the study period. These episodes are frequently associated with catheters inserted in emergency rooms, where contamination during catheter insertion is frequent [17,34]. Interestingly, most cases of CRBSI in our study were diagnosed within the first days after hospital admission and catheter insertion. The rapidity of the occurrence of these episodes was probably due to the lack of aseptic conditions at the time of insertion, while the episodes that occurred later on were associated with catheter maintenance [22]. This means that special attention should be paid to these catheters. Notably, interventions applied to reduce the incidence of CRBSI have a greater impact on episodes caused by *S. aureus* [26,27] and Gram-negative bacilli [28] than those caused by other pathogens.

The main limitation of the study is the lack of clinical information regarding the presence of chronic diseases or other health disorders that might have influenced the risk of CRBSI. In addition, CRBSI incidence rates were adjusted by patient days and not by catheter days, as this strategy would not be achievable for surveillance of all types of catheters inserted at all hospital wards, and not only in ICUs. To overcome a possible bias associated to catheter use, we estimated the incidence of CRBSI per 1,000 catheter days, which gave similar results as the adjustment by patient days. Also, due to the multicentre nature of the study, the interventions and control programmes may not have been homogeneous across the different hospitals. To overcome this limitation, the VINCat programme attempts to standardise definitions and preventive actions. In addition, the reported data are audited annually, and deviations are analysed together with the person in charge at each centre. 

## Conclusion

This surveillance programme enabled us to trace the changes in the epidemiology of CRBSI, which remains an important HAI. The present study highlights the need for interventional programmes focusing on PVC, especially in non-ICU wards. Our group is currently leading a prospective preventive programme at hospitals in Catalonia that aims to reduce the rate of CRBSI in conventional wards.

## Supplementary Data

100000 Supplement S1
