# Supplementary material for: Trends in the epidemiology of catheter-related bloodstream infections; towards a paradigm shift, Spain, 2007 to 2019
Source: Euro Surveill. 2022 May 12;27(19):2100610. doi: 10.2807/1560-7917.ES.2022.27.19.2100610 (PMC9101967; doi:10.2807/1560-7917.ES.2022.27.19.2100610)
Supplement: Supplement S1 [file 2100610_SupplementS1.pdf]

This supplementary material is hosted by *Eurosurveillance* as supporting information alongside the article [Trends in the epidemiology of catheter-related bloodstream infections; towards a paradigm shift, Spain, 2007 to 2019] on behalf of the authors who remain responsible for the accuracy and appropriateness of the content. The same standards for ethics, copyright, attributions and permissions as for the article apply. Supplements are not edited by Eurosurveillance and the journal is not responsible for the maintenance of any links or email addresses provided therein.

S1: Characteristics of hospitals participating in the VINCat program per year

Group center	2007	2008	2009	2010	2011	2012	2013	2014	2015	2016	2017	2018	2019
Group I	5	7	7	9	9	9	9	9	9	9	9	9	8
Group II	12	13	14	14	15	14	15	18	17	17	18	18	18
Group III	16	19	20	22	22	20	23	21	22	23	20	22	22
Global	33	39	41	45	46	43	47	48	48	49	47	49	48

Group I: more than 500 beds, **Group II:** between 200 and 500 beds, **Group III:** fewer than 200 beds. 20 first level hospitals, 17 second level hospitals, 17 third level university hospitals (6 of them provided with transplantation program), 27 hospitals provided with intensive care beds (Md 15 beds (IQR 9-33)]. One center is a monographic onco-haematological hospital, 19 university hospitals.
